# Sentiment analysis using averaged weighted word vector features

**DOI:** 10.1371/journal.pone.0299264

**Published:** 2024-04-04

**Authors:** Ali Erkan, Tunga Güngör

**Affiliations:** Computer Engineering Department, Boğaziçi University, İstanbul, Turkey; Infotec: Centro de Investigacion e Innovacion en Tecnologias de la Informacion y Comunicacion, MEXICO

## Abstract

People use the World Wide Web heavily to share their experiences with entities such as products, services or travel destinations. Texts that provide online feedback through reviews and comments are essential for consumer decisions. These comments create a valuable source that may be used to measure satisfaction related to products or services. Sentiment analysis is the task of identifying opinions expressed in such text fragments. In this work, we develop two methods that combine different types of word vectors to learn and estimate the polarity of reviews. We create average review vectors from word vectors and add weights to these review vectors using word frequencies in positive and negative sensitivity-tagged reviews. We applied the methods to several datasets from different domains used as standard sentiment analysis benchmarks. We ensemble the techniques with each other and existing methods, and we compare them with the approaches in the literature. The results show that the performances of our approaches outperform the state-of-the-art success rates.

## Introduction

Sentiment analysis, a pivotal subfield within natural language processing, has garnered substantial attention in recent years. This study delves into the multifaceted domain of sentiment analysis, explicitly focusing on aspect-based sentiment analysis aimed at dissecting and comprehending the polarity, nuanced emotional tones, and opinions concealed within the text. As our digital world becomes increasingly inundated with textual data, understanding the intricate subtleties of sentiment expressed by individuals in their writing has become imperative. As mentioned in Task 5 of Semeval 2016 [1], we use the Web to share our experiences about products, services or places [2]. Texts that provide online reviews are essential for consumer decision-making [3], and customer comments create valuable sources for companies to improve the satisfaction of customers. Sentiment Analysis (SA) includes several aspects of Natural Language Processing, such as entity recognition, coreference resolution, and negation handling (Liu [4] and Cambria et al. [5]) The several workshops and conferences focusing on SA-related shared tasks. Some of them are (NTCIR [6]; TAC2013 [7]; SemEval-2013 Task 2 [8]; SemEval-2014 Task 4 [9]; SemEval-2015 Task 12 [10]; SemEval-2016 Task 6 [11]; SemWebEval 2014 [12]; GESTALT-2014 [13]; SentiRuEval [14]; IberLEF-Financial Targeted Sentiment Analysis task in Spanish [15]; Evalita-ABSA task in Italian [16]. These competitions provide training datasets. Currently, most of the available SA-related datasets (Socher et al. [17]; Ganu et al. [18]) are monolingual and generally focus on English texts. Furthermore, some of them provide multilingual datasets (Klinger and Cimiano [19]; Jiménez-Zafra et al. [20]) that are useful to enable the development and testing of cross-lingual methods [21].

In addition to these, there are some other datasets, such as Stanford IMDB Reviews [22] or Yelp dataset [23], and some SA studies were made using these datasets. In this work, we focus on a comprehensive analysis of SA studies, and we are very close to the state-of-the-art results ([24, 25]).

In this work, we comprehensively analyze some existing methods to produce semantic polarities from the reviews from different domains, and we propose two new approaches to produce semantic polarities. Our approach uses weighted word vectors to create feature sets. For this purpose, we used the Word2vec [26] and the GloVe [27] models. These models were widely used in sentiment analysis [28, 29]. We ensembled and compared our approaches with existing approaches and analyzed the experimental results of IMBD movie reviews, Semeval 2016 task data set and Yelp restaurant reviews. We are very close to state-of-the-art performance accuracies with our approaches.

We provide the source code, prepare datasets for the model, and train word vectors at https://github.com/alierkan/Sentiment-Analysis.

The rest of this paper is organized as follows: The “Related Work” Section presents previous work on sentiment analysis. In the “Proposed Methodology” Section, we describe our models. In the “Experiments” Section, we describe the data sets used during the experimentation, and the results are then presented. Finally, the “Conclusion” Section summarizes this work’s conclusions and potential future directions of this work.

## Related work

In the literature, there are some datasets to use models, such as Stanford IMDB Movie Reviews, SEMEVAL Restaurant and Laptop Reviews, and the Yelp dataset. We mentioned previous studies that used these datasets. Although there are some rule-based studies in the literature, we listed only studies that used learning algorithms since our study also focuses on machine learning algorithms.

Wang and Manning [25] used support vector machine (SVM), Multinomial Naive Bayes (MNB), and SVM with NB (NBSVM) features to find out the polarities of the reviews. They split at spaces for unigrams and filtered out anything that is not [A-Za-z] for bigrams. Their approach computes a log-ratio vector between the average word counts extracted from positive documents and those from negative documents. NBSVM obtained 91.2% accuracy for the IMDB dataset.

Mesnil et al. [24] used three approaches to discriminate positive and negative sentiment for IMDB reviews, and then combined these approaches to achieve better accuracy. Their first approach is to use the Bayes rule with n-grams and Recurrent Neural Networks (RNNs) [30] to find the polarity of any review. As a second approach, they used the Naive Bayes Support Vector Machine (NB-SVM) mentioned in the previous paragraph [25]. Finally, they used a sentence vector method [31], which proposes to learn distributed representations of words and paragraphs. The sentence vector was created using the Word2vec algorithm, proposed in [26]. To create review vectors, in the first step, they added a unique ID at the beginning of each review. In this way, this id became a word that represents the review. Then, they ran the Word2vec algorithm on these modified reviews and prevented the algorithm from removing review IDs. So, they had a matrix that included word vectors of the words in the reviews and review IDs. Each row represents a word vector of the corresponding word. Then, they created a submatrix of this matrix that contains only word vectors of review IDs. This method has one major issue: A review vector should be generated from training and test reviews. Therefore, all steps, including the creation of review vectors, must be repeated to find the polarity of a new review. Therefore, it is not a practical method. Then, they combined the results of the three approaches and achieved higher accuracy. Mesnil et al. [24] passed the Wang and Manning [25] score, and reached 92.57% accuracy. In most recent studies that use IMDB reviews, Chi et al. [32] use BERT-large [33] with CNN, Qizhe et al. [34] develop an Unsupervised Data Augmentation (UDA) model with BERT and BiLSTM [35], Wang et al. [36] use RoBERTa-Large [37] with few-shot learning and Haonan et al. [38] use BERT-Large with graph neural network.

In Semeval 2016 Task 5 Subtask 2 [1], a set of customer reviews about a target entity (e.g., a laptop or a restaurant) was given; the goal is to identify a set of aspect, polarity tuples. Khalil et al.(NileTMRG Team) [39] integrated domain and aspect information into an ensemble classifier comprising three CNNs [40] trained on the complete training data from both domains. They also initialized the word vectors, which were fine-tuned using training examples collected through a semi-supervised approach within the same CNN architecture. Each of the three classifiers is similar to the one with a slight variation resulting from incorporating domain and aspect knowledge into the CNN model. They have mainly employed Static-CNN, where initialized input vectors are kept as is, and Dynamic-CNN, where input vectors are updated to optimize the network. The reviews are tokenized from the Yelp academic dataset reviews in restaurants. They ensembled their results. This ensemble model counts votes from three classifiers and predicts the class that has the maximum number of votes from the three classes, namely positive, negative, and neutral. They obtained an 85.448 percent accuracy for the English Restaurant dataset of Semeval 2016 Task 5 [1].

Kumar et al.(IIT-TUDA team) [41] used Lexical Acquisition and supervised classification using the Support Vector Machine SVM in Semeval 2016 Task 5. They used lexical expansion to induce sentiment words based on the distributional hypothesis. Due to their observation of rare words, unseen instances, and limited coverage of available lexicons, they thought that the distributional expansion might be a helpful back-off technique (Govind et al. [42]). They constructed a polarity lexicon for all languages using an external corpus and a seed sentiment lexicon. Finally, they computed normalized positive, negative and neutral scores for each word. Their primary assumption is that words with the same sentiment are semantically more similar. Hence, words that appear more in positive (negative/neutral) reviews have a higher positive (negative/neutral) sentiment score. They obtained 86.729 percent accuracy for the English Restaurant dataset of Semeval 2016 Task 5 [1].

Brun et al. (XCRE team) [43] apply a term-centric method for feature extraction. For a term, the features are obtained as the lexical-semantic categories (such as food and service) associated with the term by a semantic parser, bigrams and trigrams involving the term, and all syntactic dependencies (such as subject, object, modifier, and attribute) involving the term. First, aspects are extracted using a conditional random field (CRF) model. Then, aspect categories are found and added as features for polarity classification. The features are also delexicalized, replacing a term with its generic aspect category (e.g., “staff” is replaced by “service”, “sushi” is replaced by “food”). They obtained 88.13% accuracy for the English restaurant dataset of the Semeval 2016 Task 5 [1]. In most recent studies that use this dataset, Reddy et al. [44] use BERT with self-attention, Trusca et al. [45] use ELMo and BERT with hierarchical attention.

Jiang et al. (ECNU team) [46] employ the Logistic Regression algorithm with the default parameter implemented in lib-linear tools to build the classifiers. The 5-fold cross-validation is adopted for system development. They used linguistic features (such as Word N-grams, Lemmatized Word N-grams, and POS), sentiment lexicon features (mainly ratios between positive, negative, and potential words related to a given aspect), topic model (the document distribution among predefined topics, the topic probability of each word indicates its significance in corresponding topic), and Word2vec features to learn.

In addition to these studies that are related to the datasets that are used in this study, some current studies exist for sentiment or emotion classification. Dong et al. [47] develop a model to extract expressed and private opinions using a hierarchical network. Liu et al. [48] use the BERT encoder and the Linguistic Inquiry and Word Count (LIWC) decoder to identify emotions of reviews in a massive open online courses (MOOCs) system. Huang et al. [49] use LSTM and epistemic network analysis to explain how sentiments evolved over different levels of interaction at different stages of blended learning, which is the integration of online and classroom activities. Nie et al. [50] use a graph neural network to detect emotions in long dialogues. Ruskanda et al. [51] use variational quantum algorithms to make sentiment classification. Sadr et al. [52] use BERT and Word2Vec embedding together as input for a CNN model in which there is an attention layer before the pooling layer, and they obtained 90.97 percent accuracy for the IMDB dataset.

## Proposed methodology

By using machine learning techniques to learn the polarity of a review, we should represent it with some features. As mentioned above, they may be a bag of words, the number of words in a sentence, or other features.

Word vector models (WVMs) offer a powerful way to represent words in a continuous vector space where words with similar meanings end up close. These models have a rich history in Natural Language Processing (NLP), but they all rely on a fundamental idea known as the Distributional Hypothesis. This hypothesis suggests that words sharing similar contexts in text also share similar meanings. Approaches in WVMs can be broadly categorized into count-based and predictive methods. Count-based methods analyze how frequently a word appears alongside its neighboring words in a large text corpus and then condense this statistical information into compact vectors for each word. On the other hand, predictive models aim to predict a word based on its neighboring words, using learned compact embedding vectors as model parameters. There are two prominent word vector algorithms in this domain: Word2vec and GloVe.

Word2vec comprises a family of interconnected models designed to generate word embeddings. These models are relatively simple, consisting of two layers of neural networks, and their primary goal is to reconstruct the linguistic contexts in which words appear. The input to Word2vec is typically a sizable collection of text, from which it constructs a vector space, often with dimensions in the hundreds. Each word in the text corpus is assigned a corresponding vector within this vector space. The positioning of these word vectors is such that words sharing similar contextual surroundings in the text data are positioned close to each other within the vector space (Mikolov, 2013).

Our study utilized the Word2vec algorithm, specifically the Continuous Skip-gram Model. This model endeavors to maximize its ability to predict one word based on another word occurring in the same sentence. To achieve this, it treats each word in the text as an input to a log-linear classifier with a continuous projection layer. The model then aims to predict the words likely to appear within a specific range before and after the current word. It is important to note that expanding this range can enhance the quality of the resultant word vectors, but it also introduces increased computational complexity.

GloVe is an unsupervised learning algorithm designed to derive vector representations for words. Its training process involves analyzing collective global statistics of word co-occurrence within a corpus, and the resulting representations reveal intriguing linear relationships within the word vector space. GloVe operates by training on the nonzero entries of a global word-word co-occurrence matrix, which captures the frequency with which words appear together in a given corpus. To populate this matrix, only a single pass through the entire corpus is necessary to gather the required statistical information.

By using Word2vec and GloVe algorithms, we produced word vectors separately for the words in the reviews. That is, we have two-word vector sets for every review dataset. We obtained the best results with our methods when the lengths of the Word2vec and GloVe vectors were 300. We eliminated the stop words and did not use word vectors of the stop words. We used these vectors within our weighted averaged review vector (WARV) and the convolutional neural network (CNN) architecture described by Kim [40]. In the following sections, we will explain the methods in detail.

### Averaged Review Vector (ARV) and Weighted Averaged Review Vector (WARV)

Since word vectors represent semantic similarity between words, if we can produce a vector that represents semantic similarity for a review or sentence from word vectors, then it will be easy to learn the semantic polarity of the reviews or sentences. For that purpose, if we find a mean vector of a review from word vectors of that review, we have a new vector representing the review, which will be semantically similar to the words in the review. Therefore, we created review vectors from normalized word vectors of words in the review by averaging the word vectors. In this case, all word vectors of the words in a review will be an input of our method, and the output will be a review vector with the same dimension as word vectors. For every review, we found averaged vectors of all reviews. We produced word vectors using the Word2vec and GloVe algorithms. Then we created averaged review vectors (ARV) from Word2vec and GloVe vectors separately and concatenated them. We produced new combined word vectors from Word2vec vectors of size *M*_*W*2*V*_ and GloVe vectors of size *M*_*G*_. Obviously, the dimension of the combined word vectors is *M*_*W*2*V*_ + *M*_*G*_. For any review that contains *N* words, we have *N* word vectors with size *M*_*W*2*V*_ + *M*_*G*_: [**v**_1_, **v**_2_, …, **v**_*N*_]. Each vector (**v**_*i*_) represents a word of the review. Table 1 shows an example matrix of a review with 6 words.

**Table 1:** Embedding matrix for a review (N = 6).

Words	1	2	…	*M*_*W*2*V*_	1	2	…	*M*_*G*_
*v*_1_	-0.7731	0.0809	…	0.2855	0.0165	0.3797	…	0.2345
*v*_2_	0.4203	0.2706	…	-0.7848	-0.2526	-0.0899	…	-0.3456
*v*_3_	-0.3126	-0.4352	…	-0.5899	0.2688	0.6822	…	0.2567
*v*_4_	-0.4223	-0.8213	…	-0.8204	0.1956	-0.2388	…	0.9749
*v*_5_	-0.1547	0.0005	…	-1.0294	0.4053	-0.2716	…	-0.8065
*v*_6_	-0.6464	-0.7956	…	-0.5029	-1.1531	-0.6473	…	0.6593

Mathematically, we found normalized vectors of the word vectors by

$$\begin{array}{c} {\hat{v}}_{i}=\frac{v_{i}}{|v_{i}|} \end{array}$$ (1)

where

$$\begin{array}{c} |v_{i}|=\sqrt{v_{i1}*v_{i1}+v_{i2}*v_{i2}+...v_{i(M_{W2V}+M_{G})}*v_{i(M_{W2V}+M_{G})}} \end{array}$$ (2)

Then, we found the averaged vectors of concatenated and normalized word vectors of a review from Word2vec vectors and GloVe vectors. For that purpose, we used the following equation, which finds the average of the *N* word vectors for a review.

$$\begin{array}{c} \text{arv}=\frac{\sum_{i=1}^{N}{\hat{v}}_{i}}{N} \end{array}$$ (3)

Also, we found the weighted averaged vectors of normalized word vectors of a review from Word2vec vectors and GloVe vectors separately and concatenated them. The motivation for using weights is that if we find polar words and give them more weights, our model may learn sentiments more accurately. For that purpose, we used the following equation, which finds the weighted average of the *N* word vectors. Note that we will obtain averaged review vectors for *b*_*i*_ = 1 for every *i*.

$$\begin{array}{c} \text{warv}=\frac{\sum_{i=1}^{N}b_{i}*{\hat{v}}_{i}}{N} \end{array}$$ (4)

where *b*_*i*_ represents weights for word vectors. We used different weights and obtained the best result with the below weight for the datasets.

$$\begin{array}{c} b_{i}=\frac{P(w_{i}\geq 0)}{P(w_{i}\leq 0)} \end{array}$$ (5)

where we represented the probability of the positive polarity of the word i as *P*(*w*_*i*_ ≥ 0) and the probability of the negative polarity of the word i as *P*(*w*_*i*_ ≤ 0). To calculate these probabilities, we count the number of words in positive sentiment reviews and in negative sentiment reviews of training datasets. For example, if a word six times exists in positive reviews and four times in negative reviews, then *P*(*w*_*i*_ ≥ 0) will be 0.6 and *P*(*w*_*i*_ ≤ 0) will be 0.4. Obviously,

$$\begin{array}{c} P\left(w_{i}\geq 0\right)+P\left(w_{i}\leq 0\right)=1 \end{array}$$ (6)

**Fig 1:**
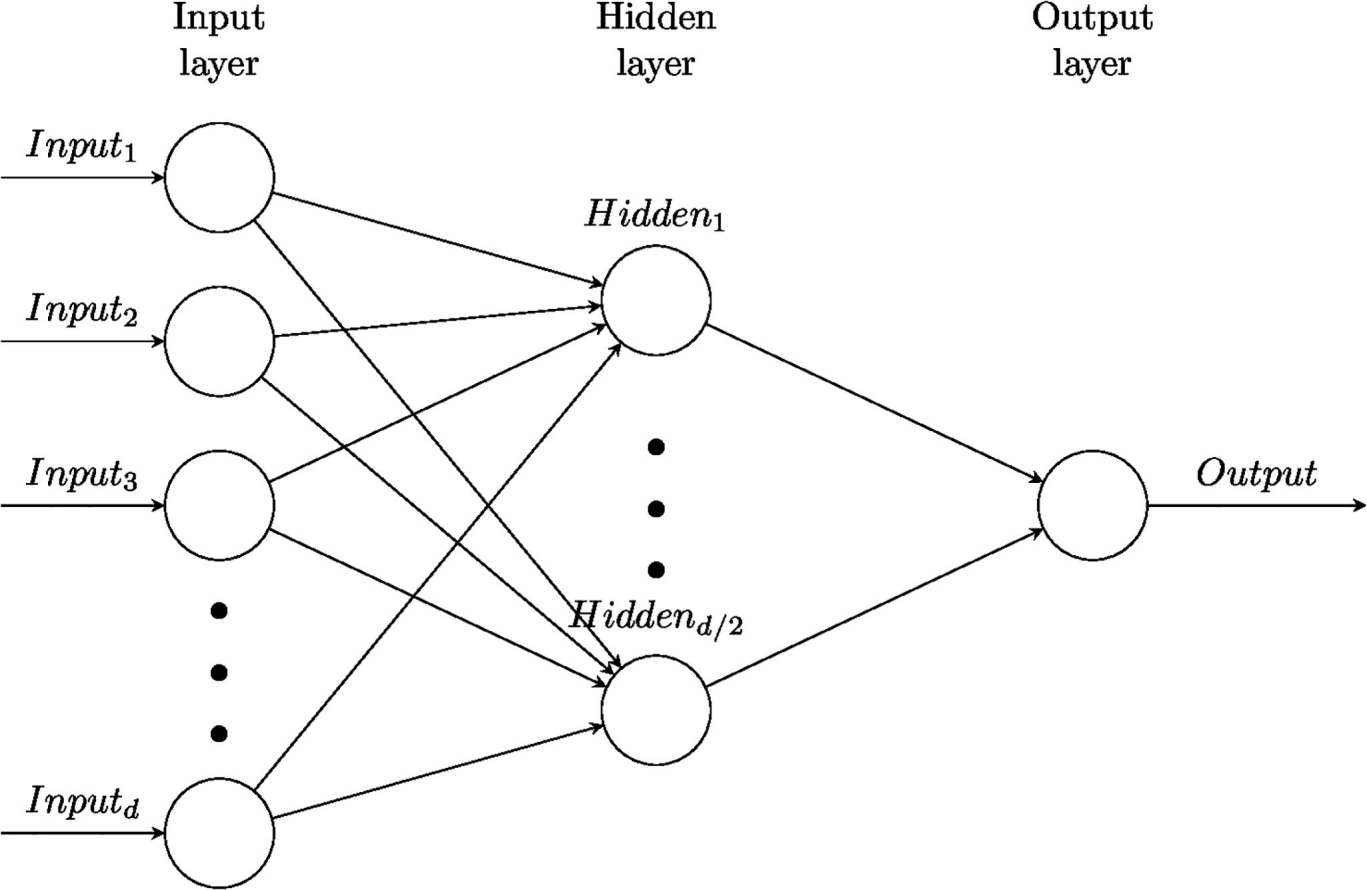
Our feed-forward neural network model.

In this way, we obtain the (weighted) averaged vectors shown in Table 2 for each review using Word2vec and GloVe vectors of the reviews as shown in Table 3. Hence, for every review, we have *M*_*W*2*V*_ + *M*_*G*_ features to learn. Using Feed-forward Neural Networks, we learned the review’s sentiment with these weighted averaged review vectors (WARV). In the next session, we shared our results for different datasets. We used the Keras framework on Tensorflow to run our feed-forward neural network model, which is shown in Fig 1. We ran our model with different numbers of hidden layers and nodes, different activation, optimizers, and loss functions; however, we obtained the best results with the following parameters: Our model includes three hidden layers. The first two layers have *M*_*W*2*V*_ + *M*_*G*_ nodes. We used the rectifier (max value of input nodes) “RELU” as an activation function. At the last layer, we used the “sigmoid” function. Our optimizer is “Adadelta,” and the loss function is “binary cross-entropy”.

**Table 2:** (Weighted) average review vectors.

Review ID	1	2	…	M_*W*2*V*_	1	2	…	M_*G*_
1	-0.7731	0.0809	…	0.2855	0.0165	0.3797	…	0.2345
2	0.4203	0.2706	…	-0.7848	-0.2526	-0.0899	…	-0.3456
3	-0.3126	-0.4352	…	-0.5899	0.2688	0.6822	…	0.2567
4	-0.4223	-0.8213	…	-0.8204	0.1956	-0.2388	…	0.9749
5	-0.1547	0.0005	…	-1.0294	0.4053	-0.2716	…	-0.8065
6	-0.6464	-0.7956	…	-0.5029	-1.1531	-0.6473	…	0.6593

**Table 3:** 2D embedding tensor for an IMDB review with 10 tokens.

No	Word	1	…	M_*W*2*V*_	1	…	M_*G*_
1	storm-lashed	0.536650	…	-0.047624	0.024453	…	0.418517
2	lended	0.182570	…	0.119859	0.203666	…	0.408951
3	maries	0.309272	…	-0.293730	0.374721	…	-0.165671
4	sinfully	0.304134	…	-0.236288	0	…	0
5	weekend	0.436948	…	0.453432	0.038075	…	-0.087220
6	bernie’s’	0.269693	…	0.545252	0.094293	…	-0.117270
7	chique	-0.238754	…	0.708557	0.062137	…	0.267371
8	spiritualistic	0.211577	…	0.226885	0.304304	…	0.006760
9	half-awake	0	…	0	-0.093967	…	0.564555
10	eddi	-0.260920	…	0.248055	-0.065446	…	-0.259685
…	…	0	…	0	0	…	0
N	…	0	…	0	0	…	0

### Convolutional Neural Network (CNN)

**Fig 2:**
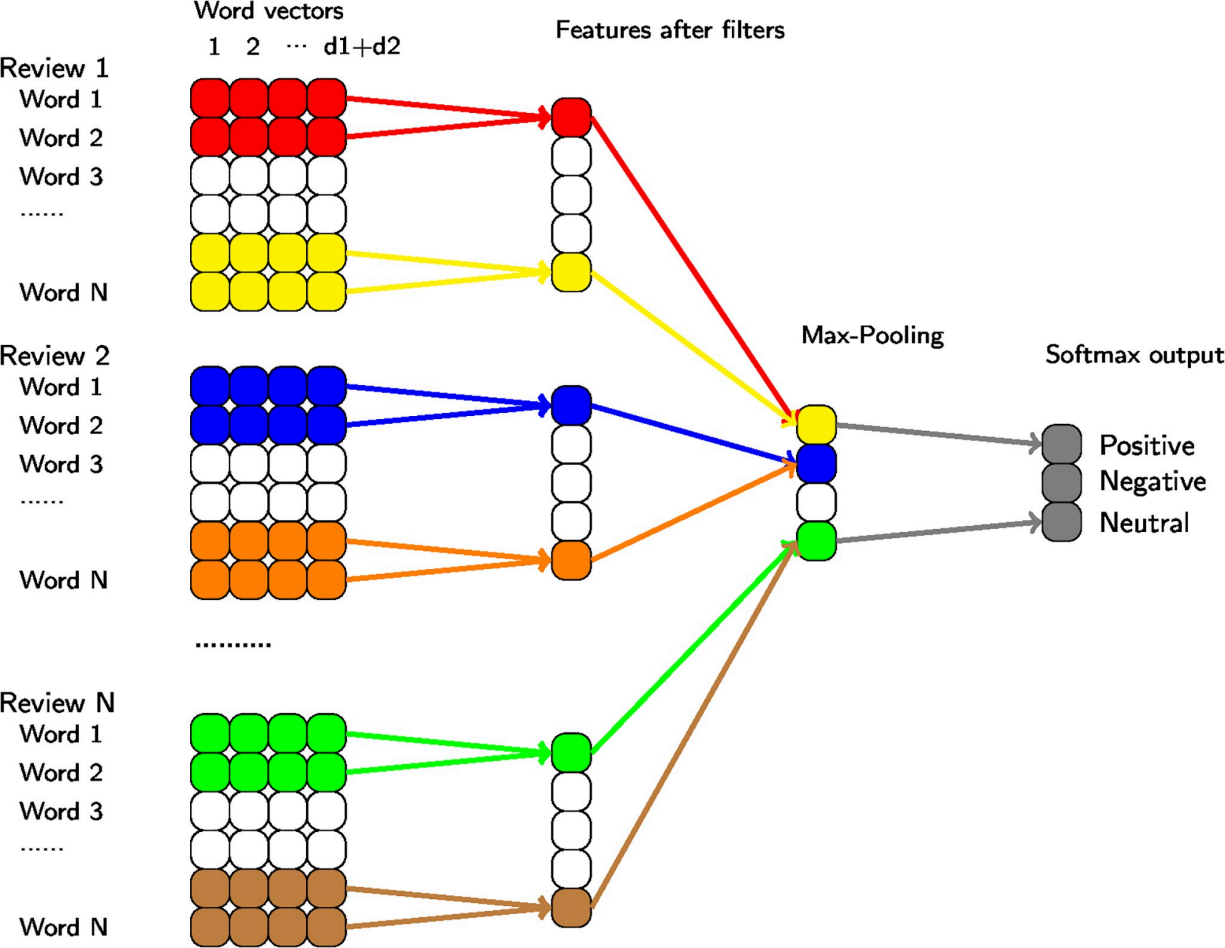
CNN sentence classification.

We used Convolutional Neural Networks to learn the sentiment of the reviews by using Word2vec vectors and GloVe vectors shown in Table 3. We used word vectors as features directly. Our learning system is based on the Deep Convolutional Neural Network (CNN) architecture described by Kim [40]. Our architecture is shown in Fig 2.

A review matrix is built for each input review, where each row is a vector representation of the word in the review. The review length is fixed to the maximum length of the dataset so that all review matrices have the exact dimensions. (Shorter reviews are padded with row vectors of 0s accordingly.) Each row vector of the review matrix comprises columns corresponding to Word2vec and GloVe vectors concatenated together. For every word in the review, one embedding vector is used, and all vectors are concatenated for each review. In this way, we obtained a two-dimensional tensor as input of the CNN. Additionally, padding vectors are added for reviews whose tokens are less than the tokens of the largest review. Then, a set of filters is applied to these input tensors of all reviews to produce a feature map.

Next, we employ a max-over-time pooling operation over the feature map, a technique introduced by Collobert et al. (2011) [53]. This operation entails identifying the maximum value within the feature map, denoted as $\hat{c}=\text{max}\;c$. The underlying concept is to capture the most significant feature in each feature map by selecting the one with the highest value. This pooling method naturally accommodates varying lengths of reviews, ensuring that one feature is extracted from each filter. The model incorporates multiple filters with different window sizes to derive a range of distinct features. These features collectively constitute the penultimate layer of the model and are subsequently passed to a fully connected softmax layer. The output of this softmax layer represents the probability distribution over different labels, enabling the model to make classifications based on the learned features.

For every review, we produced review matrices whose rows represent word vectors obtained by concatenation of Word2vec and GloVe as shown in Table 3. If one word does not exist in Word2vec/GloVe vectors, then we fill into cells corresponding to Word2vec/GloVe columns with zeros. Also, the number of rows of the review matrices is fixed to some words of maximum-length review (N). For the reviews with fewer words than N, empty rows are filled with zeros. Our Word2vec and GloVe vectors are 300; therefore, we have an input vector whose length is 600 (= 300 + 300).

We used the Keras/Tensorflow [54] framework to run CNN. In this framework, by default, the filters are initialized randomly using the glorot uniform method. Again, we used 600 filters, equal to the input’s dimension. At each layer of the network, these filters are applied individually.

During the training process, the values within these filters are optimized using backpropagation, a common technique in deep learning, leveraging a specific loss function. In our study, we employed the binary cross-entropy loss for tasks that involve positive and negative sentiment classification (utilizing the IMDB dataset as a reference [22]). We used the categorical cross-entropy loss function for cases where the sentiment analysis task included neutral sentiment as well, as seen in the Semeval 2016 dataset [1].

### Ensemble

After learning using training data, we combine the results of different learning algorithms using validation data sets. We used two different ensemble approaches: As a first ensemble approach (Ensemble-1), we used the log probability scores approach in Mesnil’s study [24]. In this approach, instead of directly combining the class labels predicted by each model, the logarithm of probabilities corresponding to each class are aggregated across all models by weighted averaging. Weights are determined experimentally. Once the aggregated log probabilities are computed, they are transformed back into probabilities using the exponential function.

**Fig 3:**
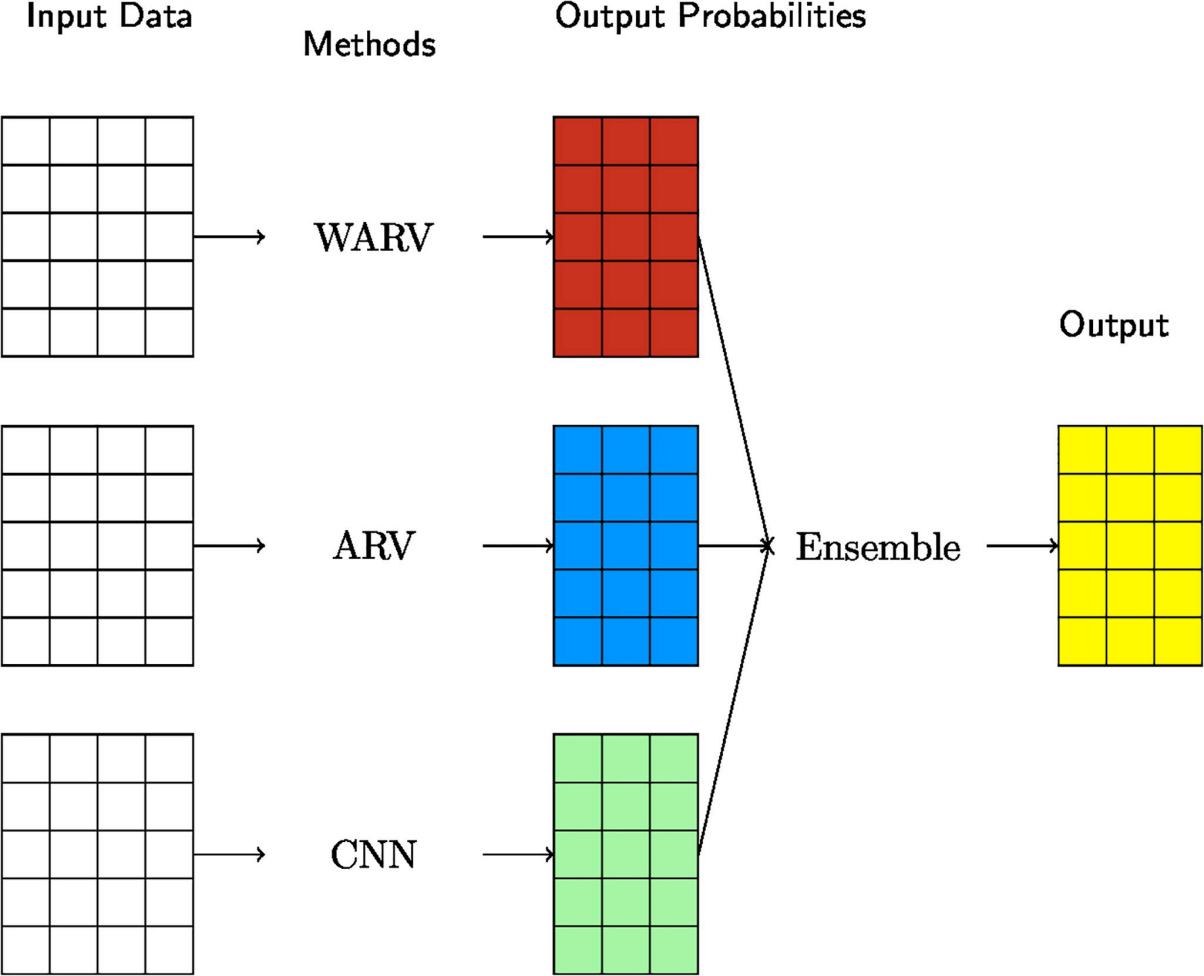
Ensemble of learning algorithms.

As the second one, we used a neural network over validation sets (Ensemble-2) in Fig 3. In this case, our learning algorithms produce results by using validation data sets. The results contain probabilities for each class. Then, we used these results as features of the ensemble learning algorithm. For that purpose, we used different learning algorithms to ensemble the methods, and we obtained the best accuracies with logistic regression and neural networks. Our neural network model contains three hidden layers whose number of nodes is equal to the input length. We used Rectified Linear Unit as the activation function of the layers, Sigmoid function to estimate the class or label, AdaDelta [55] as an optimizer, and cross-entropy as a loss function. Then we tested our ensemble methods with test datasets again. Our ensemble method produced better results than every single learning algorithm.

## Experiments

This work tests our models and other existing models with different datasets. We used the Stanford IMDB Reviews [22], SEMEVAL 2016 Task 5 dataset [1] and the YELP dataset [23]. In this section, we explain experiments and results. Table 4 shows the used hyper-parameters in our models. Learning rate and epsilon are the parameters of the “Adadelta” optimizer. Experiments run on Nvidia RTX 3090 GPU.

**Table 4:** Values of hyper-parameters.

Parameter	Value
Batch Size	32
Number of Epochs	100
Learning Rate	0.001
Epsilon	1e-7
Dropout ratio	0.3

### IMDB reviews

The Stanford IMDB review dataset contains 100,000 movie reviews in English. 25,000 reviews are labeled as positive, the other 25,000 are labeled as negative, and the remaining 50,000 are unlabeled. IMDB reviews are labeled numbers that are between 1 and 10. However, this dataset contains only reviews with 1 to 4 as negative and reviews with 7 to 10 as positive. Therefore, there are only two labels: positive and negative. For IMDB reviews, we produced Word2vec and GloVe vectors from IMDB reviews. The length of the vectors is 300, and their window sizes are 5. We run our methods using Word2vec vectors(_wv) and GloVe vectors(_gl) separately and combined(_wvgl). Therefore, the number of features of our Averaged Review Vectors (ARV) and Weighted Averaged Review Vectors (WARV) is 300 for separate runs and 600 for combined runs. Similarly, for our CNN model, the number of features is 300 (separate run) and 600 (combined run).

#### McNemar’s test

McNemar’s test [56] is usually used to determine if the success rates of two different algorithms when running on the same input data set are significantly different from each other. It is applied to 2×2 contingency tables. We used this test to compare the results of the methods to check whether both methods produced significantly different results. For this purpose, we compared the methods two by two, and the results are listed in Table 5. As seen in the table, all methods produce significantly different results from each other at the significance level *p* = 0.01. There is only one exception for the RNNLM and CNN methods. Therefore, we may conclude that especially our WARV method improves the accuracy significantly.

**Table 5:** McNemar’s test results of the methods.

Method 1	Method 2	Test Statistic	p-value
RNNLM	PARAGRAPH	43.513	0.000
RNNLM	NBSVM	9.948	0.002
RNNLM	ARV	258.930	0.000
RNNLM	CNN	1.099	0.295
PARAGRAPH	NBSVM	19.931	0.000
PARAGRAPH	ARV	156.272	0.000
PARAGRAPH	CNN	86.196	0.000
NBSVM	ARV	265.454	0.000
NBSVM	CNN	27.795	0.000
ARV	CNN	429.937	0.000
WARV	RNNLM	89.331	0.000
WARV	PARAGRAPH	8.853	0.003
WARV	NBSVM	66.513	0.000
WARV	ARV	77.704	0.000
WARV	CNN	155.252	0.000

#### Ensemble

As seen in Table 6, with **% 94.95** accuracy, our WARV method has better accuracy values than the N-Gram, RNNLM and Mesnil’s paragraph vector. Also, our WARV method is computationally more efficient than Mesnil’s paragraph vectors (PV) (Mesnil et al. [24]).

**Table 6:** Accuracies of methods before ensemble for IMDB dataset.

Methods	Accuracies	Explanation
RNNLM	86.30	Recurrent Neural Network (Mesnil et al. [24])
PV	88.45	Paragraph Vectors (Mesnil et al. [24])
NBSVM	91.87	NB-SVM TriGram (Wang et al. [24, 25])
CNN	**95.79**	BERT-Large [32]
BiLSTM	**95.8**	BERT + UDA [34]
Few-shot learning	**96.1**	RoBERTa-Large [36]
Graph Neural Net	**96.0**	BERT-Large [38]
ACNN-TL	**90.97**	Attention CNN with BERT and Word2Vec [52]
ARV_wv	88.05	Average Vectors of Reviews with Neural Network (Word2vec)
ARV_gl	84.54	Average Vectors of Reviews with Neural Network (GloVe)
ARV	88.75 ± 0.38	Average Vectors of Reviews with Neural Network (Word2vec + GloVe)
WARV_wv	93.91	Weighted Average Vectors of Reviews with Neural Network (Word2vec)
WARV_gl	86.24	Weighted Average Vectors of Reviews with Neural Network (GloVe)
WARV	**94.95** ± 0.08	Weighted Average Vectors of Reviews with Neural Network (Word2vec + GloVe)
CNN	89.19	Convolutional Neural Network with Word Vectors

When our weighted averaged review vectors (WARV) are ensembled with NBSVM, they reach the best accuracy value **% 95.56** is close to the state-of-the-art result **% 96.10** [36] with RoBERTa-Large embedding as shown in Tables 6 and 7 respectively. When we ensemble all six methods, accuracy becomes **% 95.48**. These results are nearly close to other results that were obtained by using Bidirectional Transformers BERT embeddings [33] in some studies [32, 34, 36, 38] that reached **% 96.10** [36].

**Table 7:** Accuracies of methods after ensemble for IMDB dataset.

Methods	Ensemble-1	Ensemble-2
RNNLM, PARAGRAPH	90.14 (Mesnil et al. [24])	86.98 ∓ 1.18
RNNLM, NBSVM	92.08 (Mesnil et al. [24])	91.03 ∓ 0.39
PARAGRAPH, NBSVM	92.13 (Mesnil et al. [24])	91.80 ∓ 0.43
WARV, RNNLM	95.22 ∓ 0.06	94.76 ∓ 0.21
WARV, PARAGRAPH	94.62 ∓ 0.04	94.70 ∓ 0.09
WARV, NBSVM	**95.56 ∓ 0.03**	94.92 ∓ 0.54
WARV, ARV	94.35 ∓ 0.09	94.70 ∓ 0.23
WARV, CNN	94.44 ∓ 0.07	93.82 ∓ 1.18
RNNLM, PARAGRAPH, NBSVM	92.37 (Mesnil et al. [24])	91.92 ∓ 0.28
WARV, PARAGRAPH, NBSVM	94.59 ∓ 0.02	95.42 ∓ 0.13
WARV, PARAGRAPH, ARV	93.25 ∓ 0.07	94.93 ∓ 0.17
WARV, PARAGRAPH, CNN	93.42 ∓ 0.04	94.99 ∓ 0.22
WARV, NBSVM, ARV	94.45 ∓ 0.06	94.82 ∓ 1.33
WARV, NBSVM, CNN	94.10 ∓ 0.07	95.02 ∓ 0.90
WARV, ARV, CNN	93.37 ∓ 0.07	95.07 ∓ 0.11
RNNLM, PARAGRAPH, WARV	94.59 ∓ 0.04	94.85 ∓ 0.15
RNNLM, WARV, ARV	94.42 ∓ 0.07	94.82 ∓ 0.21
RNNLM, WARV, CNN	94.22 ∓ 0.06	94.94 ∓ 0.15
RNNLM, NBSVM, WARV	95.18 ∓ 0.02	95.34 ∓ 0.26
RNNLM, PARAGRAPH, NBSVM, WARV	94.47 ∓ 0.03	95.35 ∓ 0.16
RNNLM, PARAGRAPH, ARV, WARV	93.50 ∓ 0.07	94.78 ∓ 0.28
PARAGRAPH, NBSVM, ARV, WARV	93.85 ∓ 0.04	95.31 ∓ 0.18
PARAGRAPH, NBSVM, CNN, WARV	93.89 ∓ 0.04	94.68 ∓ 1.27
PARAGRAPH, WARV, ARV, CNN	93.14 ∓ 0.05	95.11 ∓ 0.06
RNNLM, NBSVM, ARV, WARV	94.45 ∓ 0.06	95.42 ∓ 0.12
RNNLM, NBSVM, CNN, WARV	94.26 ∓ 0.04	95.24 ∓ 0.08
NBSVM, WARV, ARV, CNN	93.92 ∓ 0.05	95.38 ∓ 0.19
RNNLM, WARV, ARV, CNN	93.70 ∓ 0.07	95.06 ∓ 0.09
PARAGRAPH, WARV, ARV, CNN	93.14 ∓ 0.05	94.65 ∓ 0.71
WARV, PARAGRAPH, NBSVM, ARV, CNN	93.57 ∓ 0.05	95.30 ∓ 0.25
RNNLM, WARV, NBSVM, ARV, CNN	94.06 ∓ 0.04	95.38 ∓ 0.10
RNNLM, PARAGRAPH, WARV, ARV, CNN	93.35 ∓ 0.05	95.09 ∓ 0.06
RNNLM, PARAGRAPH, NBSVM, WARV, CNN	94.04 ∓ 0.03	95.37 ∓ 0.15
RNNLM, PARAGRAPH, NBSVM, ARV, WARV	93.91 ∓ 0.04	95.29 ∓ 0.15
RNNLM, PARAGRAPH, NBSVM, ARV, CNN, WARV	93.68 ∓ 0.05	95.48 ∓ 0.04

Note that we did not share the ensemble results of the combinations whose accuracies are below % 94 except for ensembles in Mesnil’s study [24].

### Semeval 2016 Task 5 dataset

In English, two domain-specific datasets for consumer electronics (laptops) and restaurants, consisting of more than 1000 review texts (approximately 6K sentences) with fine-grained human annotations (opinion target expressions, aspect categories, and polarities) will be provided for training/development. In particular, the SE-ABSA15 train and test datasets for restaurants and laptops (with some corrections) will be made available as training data. They consist of 800 review texts (4500 sentences) annotated with approximately 15000 unique label assignments (Entity, Aspect, Polarity). The laptop dataset comprises 450 review texts (2500 sentences) annotated with 2923 Entity#Aspect, polarity tuples. The restaurant dataset comprises 350 review texts (2000 sentences) annotated with 2499 Entity#Aspect, polarity tuples. All datasets will be enriched with text-level annotations. In addition, data sets exist for other languages rather than English. These languages are Arabic, Chinese, Dutch, French, Russian, Spanish, and Turkish [1].

We used our two methods in the English restaurant dataset. We produced Word2vec and GloVe vectors from the Yelp dataset. For this purpose, we first get only restaurant-related reviews from the Yelp dataset and then create word vectors. After that, we applied our two methods (ARV and CNN) to the Semeval 2016 Task 5 dataset to learn polarity. Mainly, our ARV method produced high accuracy results (**87.7%**). It is very close to the best result of Semeval 2016 Task 5 as shown in Table 8 and the study [44] (**87.8%**) with BERT embedding [33].

**Table 8:** Accuracies of methods (Ensemble-2) for semeval restaurant dataset.

Methods	Accuracies	Explanation
Self Attention	**88.70**	BERT-IL [44]
**ARV, WARV and CNN**	**88.669 ∓ 3.60**	Ensemble of ARV, WARV and CNN by Neural Network
**WARV and CNN**	**88.630 ∓ 3.81**	Ensemble of WARV and CNN by Neural Network
**ARV and CNN**	**88.475 ∓ 1.79**	Ensemble of ARV and CNN by Neural Network
XRCE	88.126	Semeval 2016 Task 5 Slot 1 Best Accuracy [1]
Hierarchical Attention	87.00	ELMo + BERT [45]
IIT-T	86.729	Semeval 2016 Task 5 Slot 1 Second Best Accuracy [1]
NileT	85.448	Semeval 2016 Task 5 Slot 1 Third Best Accuracy
IHS-R	83.935	Semeval 2016 Task 5 Slot 1 Fourth Best Accuracy [1]
ECNU	83.236	Semeval 2016 Task 5 Slot 1 Fifth Best Accuracy [1]
**WARV**	**83.95 ∓ 0.38**	Weighted Averaged Review Vectors with Neural Network
**CNN**	**83.120 ∓ 0.44**	Convolutional Neural Network with Word Vectors
INSIG	82.072	Semeval 2016 Task 5 Slot 1 Sixth Best Accuracy [1]
**ARV**	**82.08 ∓ 0.68**	Averaged Review Vectors with Neural Network
**basel**	76.484	Base value of Semeval 2016 Task 5 Slot 1 [1]

We ensembled our two results using a neural network. For this purpose, we used the probability output of ARV and CNN. Since we have three classes (positive, neutral, and negative), each method has three probability values. Therefore, we used six probability values of two methods as our learning features. Since the Semeval 2016 dataset is tiny, we have no opportunity to use some parts of the dataset for validation. Instead of probability values in the validation data, we used probabilities of the training dataset. Our ensemble feed-forward neural network used probabilities produced over the training dataset of Semeval 2016 Task5 by our ARV and CNN methods. Then we used the test dataset of Semeval 2016 Task 5 to evaluate our ensemble model. As shown in Table 8, our ensemble produced results that are very close to the state-of-the-art value (**88.242%**) for the Semeval 2016 Task 5 dataset. We used the Keras framework to run our ensemble neural network model. Our ensemble learning neural network includes three hidden layers with 600 nodes, equal to the number of features. As an activation function, we used “scaled exponential linear units” (SELUs), which induce self-normalizing properties [57]. At the last layer, we used the “softmax” function. Our optimizer is “Adadelta,” and the loss function is “binary cross-entropy”.

Note that we did not share the results of the RNNLM, NBSVM and PV methods, which were compared with our methods in the previous section for this dataset since they suffered from a small dataset. They could not learn accurately, and their results could have been better.

Pretrained transformer-based models have recently been used in sentiment analysis [58]. We also used average pretrained transformer-based models BERT vectors [33] to compare our results with transformer-based models. For that purpose, we got vector tokens in the last layer of the BERT model; then, we calculated the average for each review, which is the same method as our other models. Then, we used these vectors instead of our Word2vec and GloVe vectors to learn the sentiment of the reviews. For the Semeval 2016 dataset, we obtained 86.39% accuracy which is less than our ensemble results. Furthermore, for the IMDB movie review dataset, we obtained 90.43% accuracy, which is less than our ensemble results.

### Yelp dataset

We tested a weighted average review vector with randomly chosen restaurant-related reviews. When we used 100.000 reviews (50.000 positive, 50.000 negative) for training and 100.000 reviews (50.000 positive, 50.000 negative) for testing, our accuracy was % 73.811. When we used 500.000 reviews (250.000 positive, 250.000 negative) for training and 500.000 reviews (250.000 positive, 250.000 negative) for testing, our accuracy was % 77.496. When we randomly selected 100.000 restaurant reviews (50.000 positives, 50.000 negatives) and divided the data into 90% for training and 10% for testing, we applied a ten-fold cross-validation. As seen in Table 9, ensembling does not increase the accuracies for the randomly chosen Yelp dataset. This means that all methods are able to learn the same reviews correctly and make mistakes for the same reviews.

**Table 9:** Accuracies for Yelp restaurant dataset with 100.000 reviews.

Methods	Accuracies	Explanation
ARV, WARV and CNN	75.51 ∓ 0.53	Ensemble of ARV, WARV and CNN by Neural Network
WARV and CNN	74.55 ∓ 0.42	Ensemble of WARV and CNN by Neural Network
ARV and CNN	75.12 ∓ 0.37	Ensemble of ARV and CNN by Neural Network
ARV and WARV	75.80 ∓ 0.67	Ensemble of ARV and CNN by Neural Network
CNN	73.22 ∓ 0.90	Convolutional Neural Network with Word Vectors
WARV	75.76 ∓ 0.19	Weighted Averaged Review Vectors with Neural Network
ARV	75.89 ∓ 0.39	Averaged Review Vectors with Neural Network

We produced word2vec and glove vectors with dimensions 200 for each using a window size of five. We run our feedforward models using word2vec vectors and glove vectors together, and we got the best results with dimension 200. Thus, the number of features in the models is 200 for the separate runs and 400 for the combined runs. Similarly, in the CNN model, the column dimensions of the input matrices are 200 (separate run) and 400 (combined run).

Again, we used word vectors that were produced from the Yelp dataset. This dataset [23] is a subset of reviews and user data, especially for use in academic purposes. Available in both JSON and SQL files. Each file comprises a single object type, one JSON object per line. According to the business IDs, we divided the review file into two separate files; one of them consists of restaurant reviews and the other one consists of non-restaurant reviews. For restaurants, we used approximately two million reviews to produce word vectors. For this purpose, we used two files from the Yelp dataset:

- business.json: Business data with location data, attributes, and categories.
- review.json: Review text data.

## Conclusion

In this work, we used a combination of Word2vec and GloVe vectors. These vectors were used directly for the CNN model to create review matrices. For our averaged vector model, for a review, we found averaged vectors from word vectors of the word in the review. For our weighted averaged vector model, we first multiplied the word vectors by predefined values, such as the ratio of numbers of positive and negative words. Then, we found averaged vectors. Then, we used these vectors as input to the learning algorithms. We compared our results with different methods and with different datasets.

Moreover, we are very close to the state-of-the-art results for the IMDB dataset and the Semeval-2016 Task-5 dataset although we did not use any pre-trained model such as BERT [33] or the Large Language Model LLAMA [59]. Our weighted averaged vector model obtained high accuracy values for the IMDB dataset and is computationally very efficient. Building word vectors only once is enough for our method, although the paragraph vector method needs to build word vectors for all new input data. Furthermore, our ensemble results are very high, and our ensemble method produced better results than every single learning algorithm. For our method, the next step is a lookup operation to find the word vector of the words and calculate the weighted average of the word vectors. We will continue to evaluate our models and their ensembles with different datasets in different domains. Furthermore, our models do not include language-specific features. Therefore, we will test our model in different languages rather than English. In addition, we will find different weights that may produce better results.

There may be some potential directions for future research on sentiment analysis. We make experiments with different weighting schemes to enhance the effectiveness of combining Word2Vec and GloVe embeddings. We implement attention mechanisms to add dynamic weights to the word embeddings within the averaging process. We use other word embeddings or contextual embeddings such as BERT and investigate how combining multiple types of embeddings could improve sentiment analysis performance.

## Supporting information

S1 File (ZIP)
